# Supplementary figures and images for: The relationship of work engagement with job experience, marital status and having children among flexible workers after the Covid-19 pandemic
Source: PLoS One. 2022 Nov 11;17(11):e0276784. doi: 10.1371/journal.pone.0276784 (PMC9651564; doi:10.1371/journal.pone.0276784)

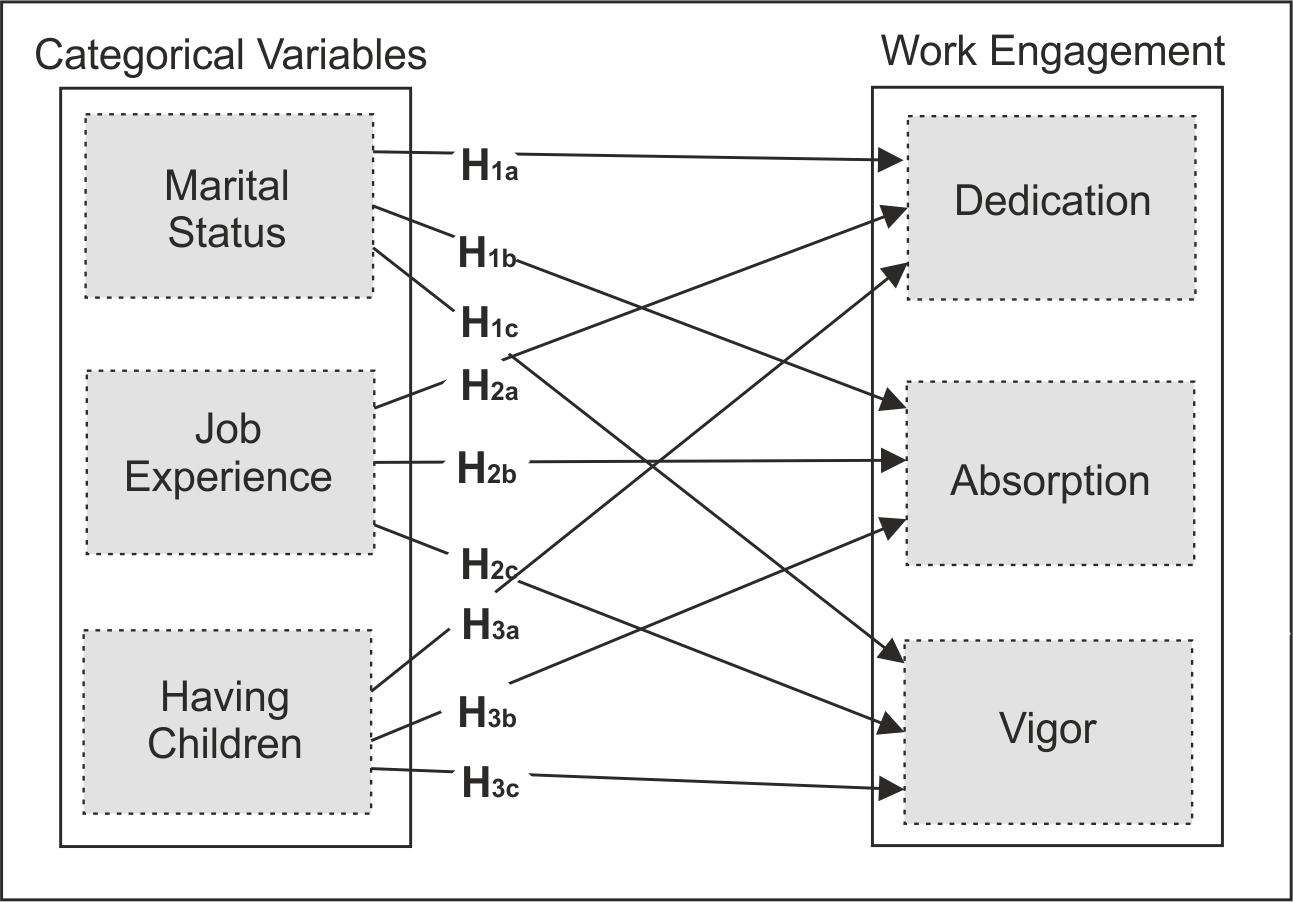

Supplement: S1 Fig — (TIF) [file pone.0276784.s001.tif]

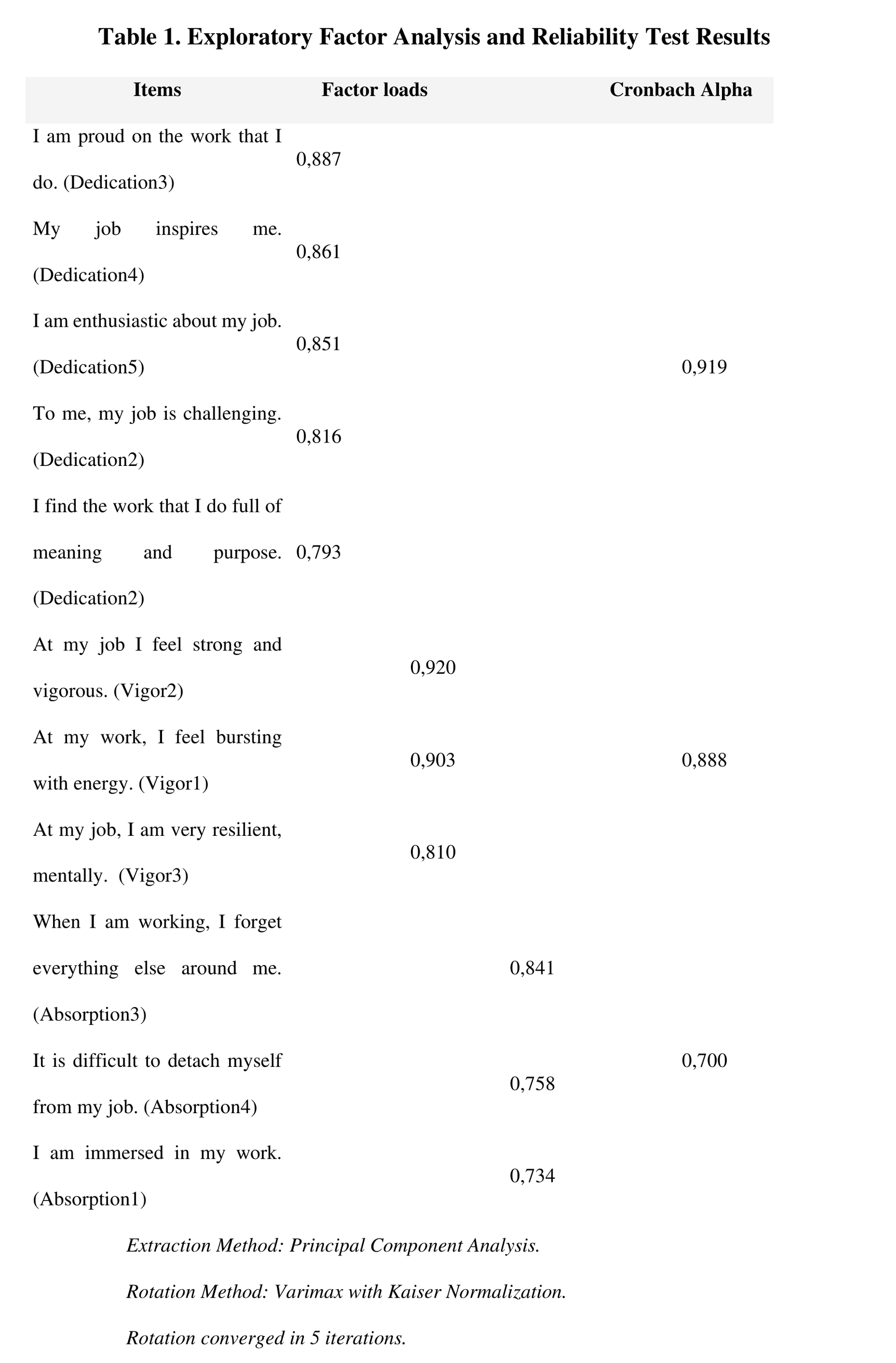

Supplement: S1 Table — (TIF) [file pone.0276784.s002.tif]

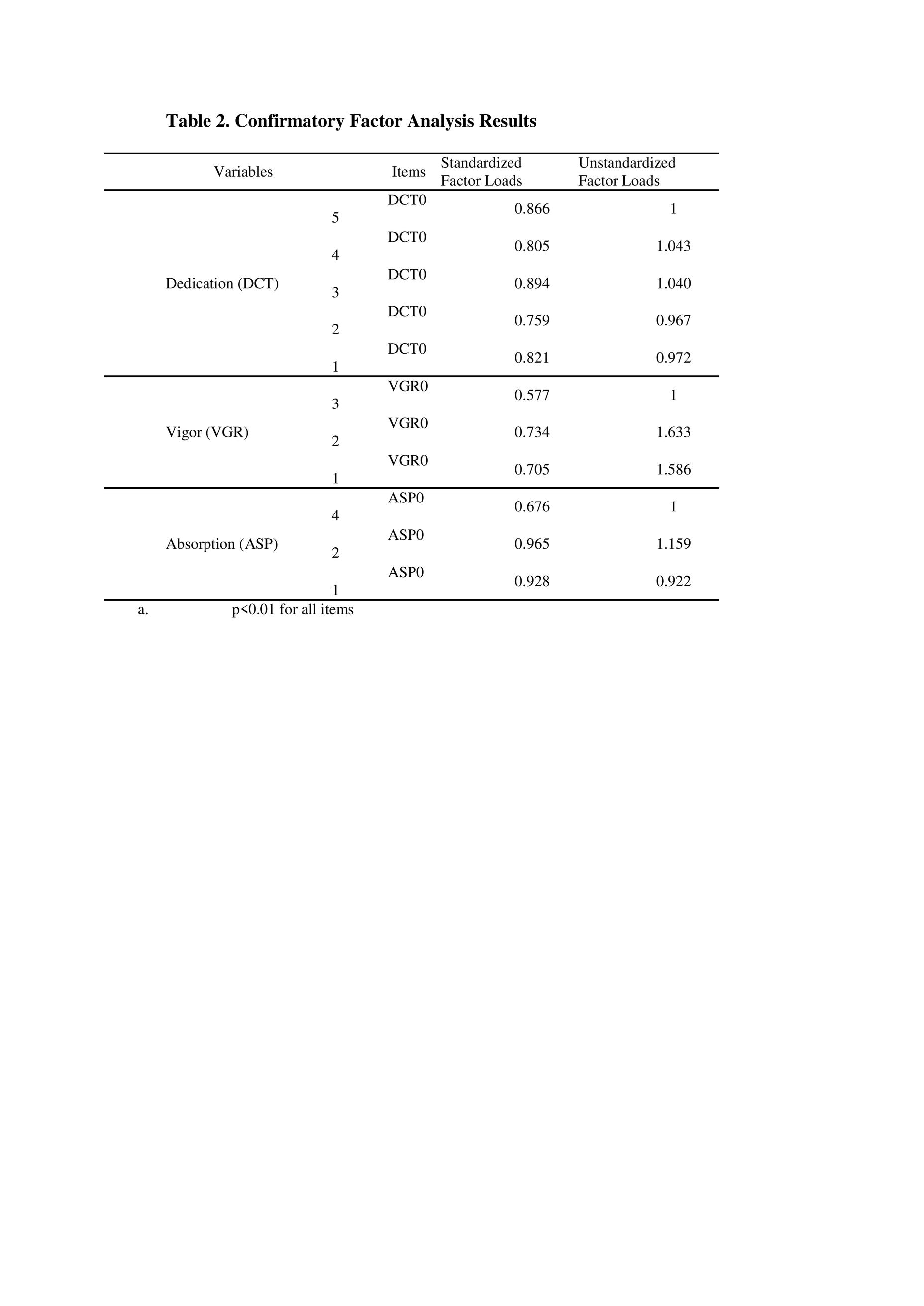

Supplement: S2 Table — (TIF) [file pone.0276784.s003.tif]

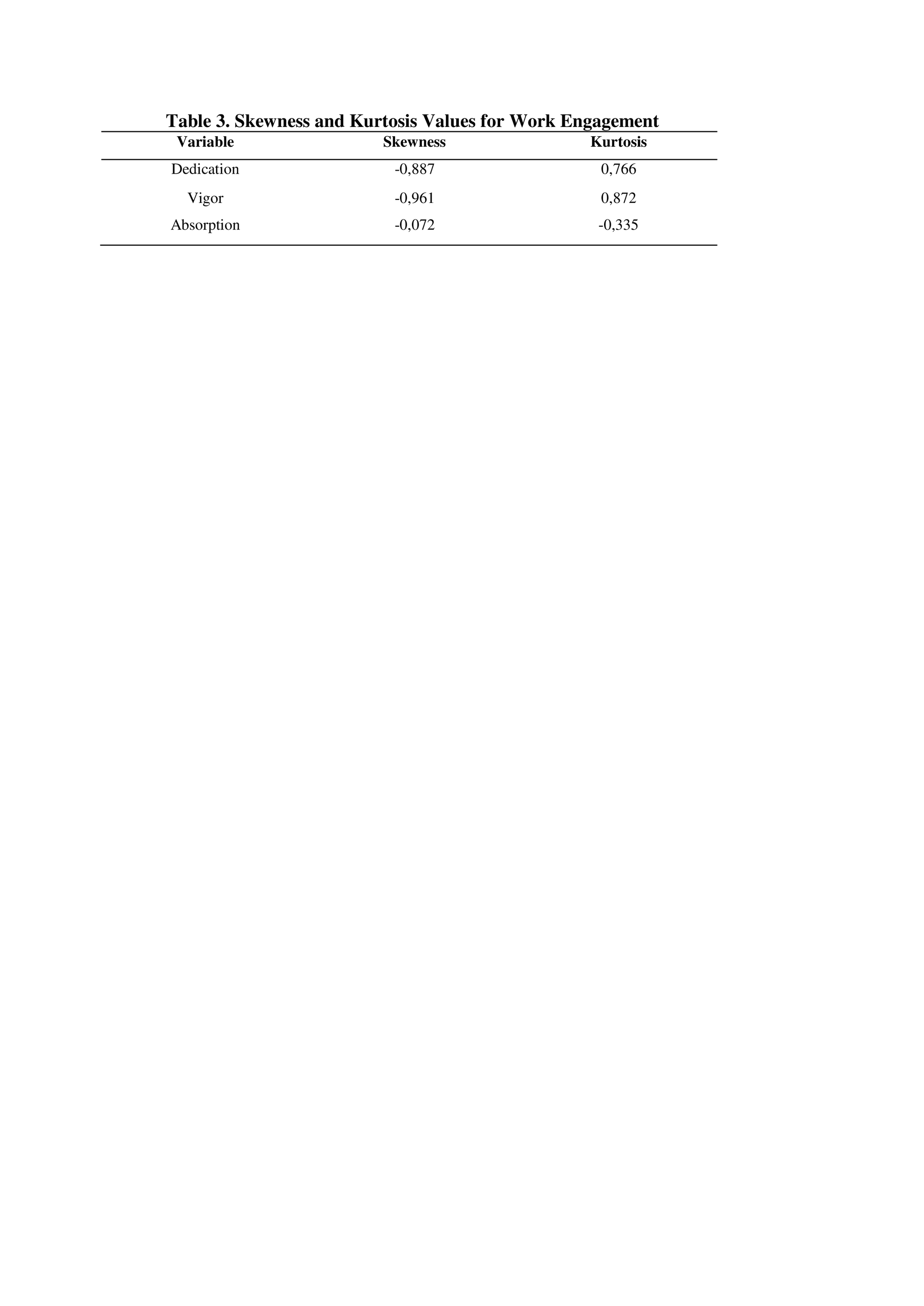

Supplement: S3 Table — (TIF) [file pone.0276784.s004.tif]

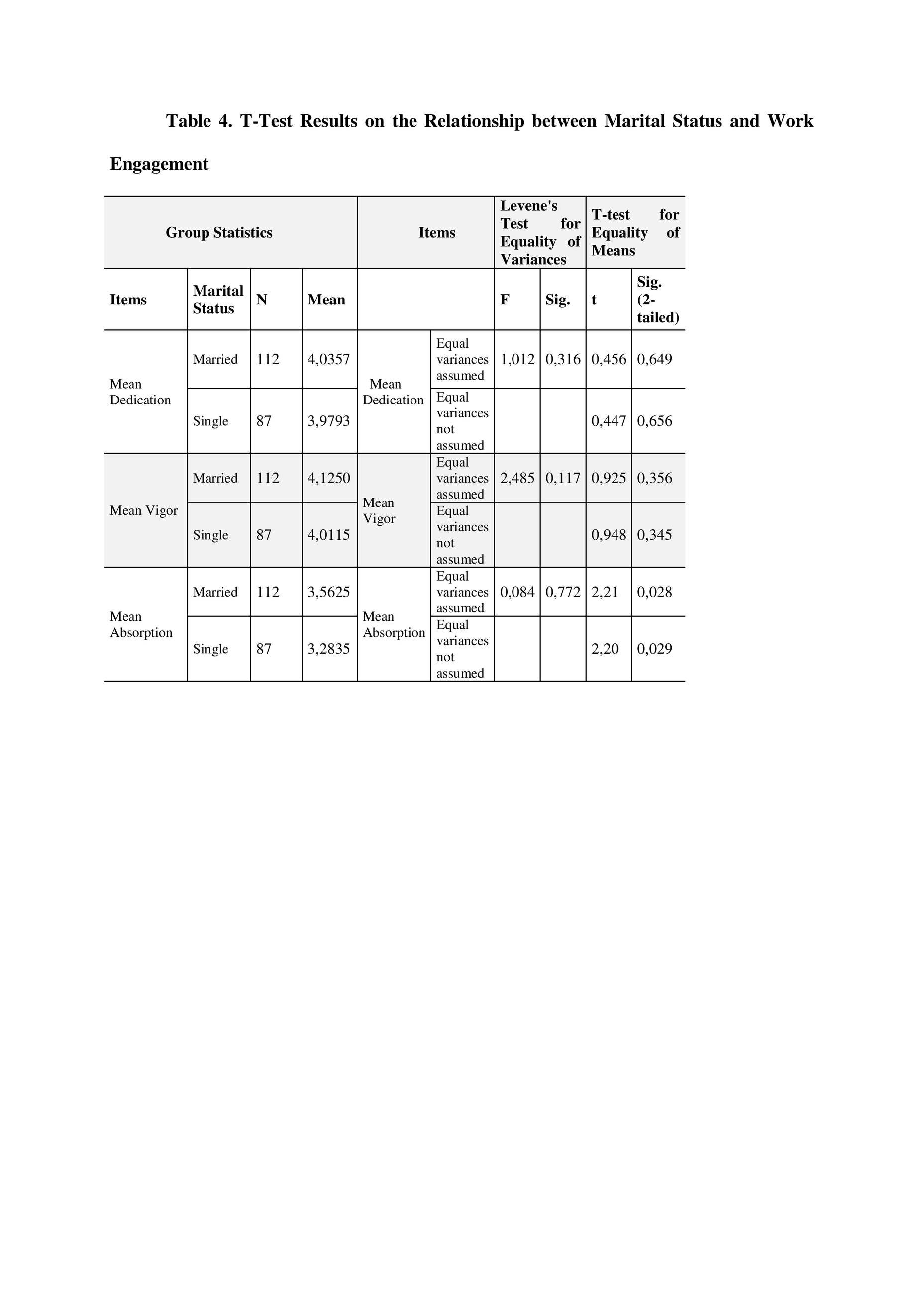

Supplement: S4 Table — (TIF) [file pone.0276784.s005.tif]

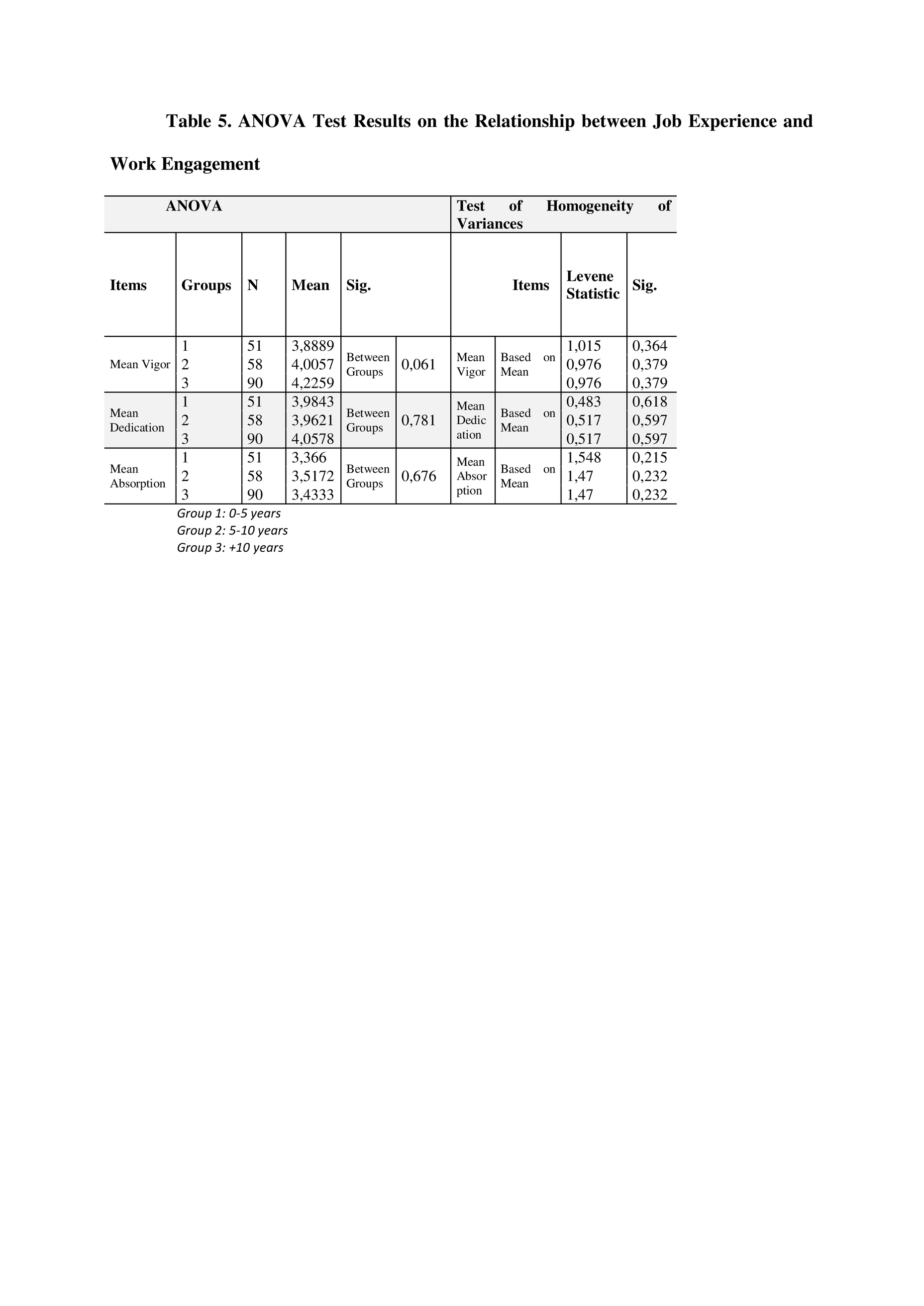

Supplement: S5 Table — (TIF) [file pone.0276784.s006.tif]

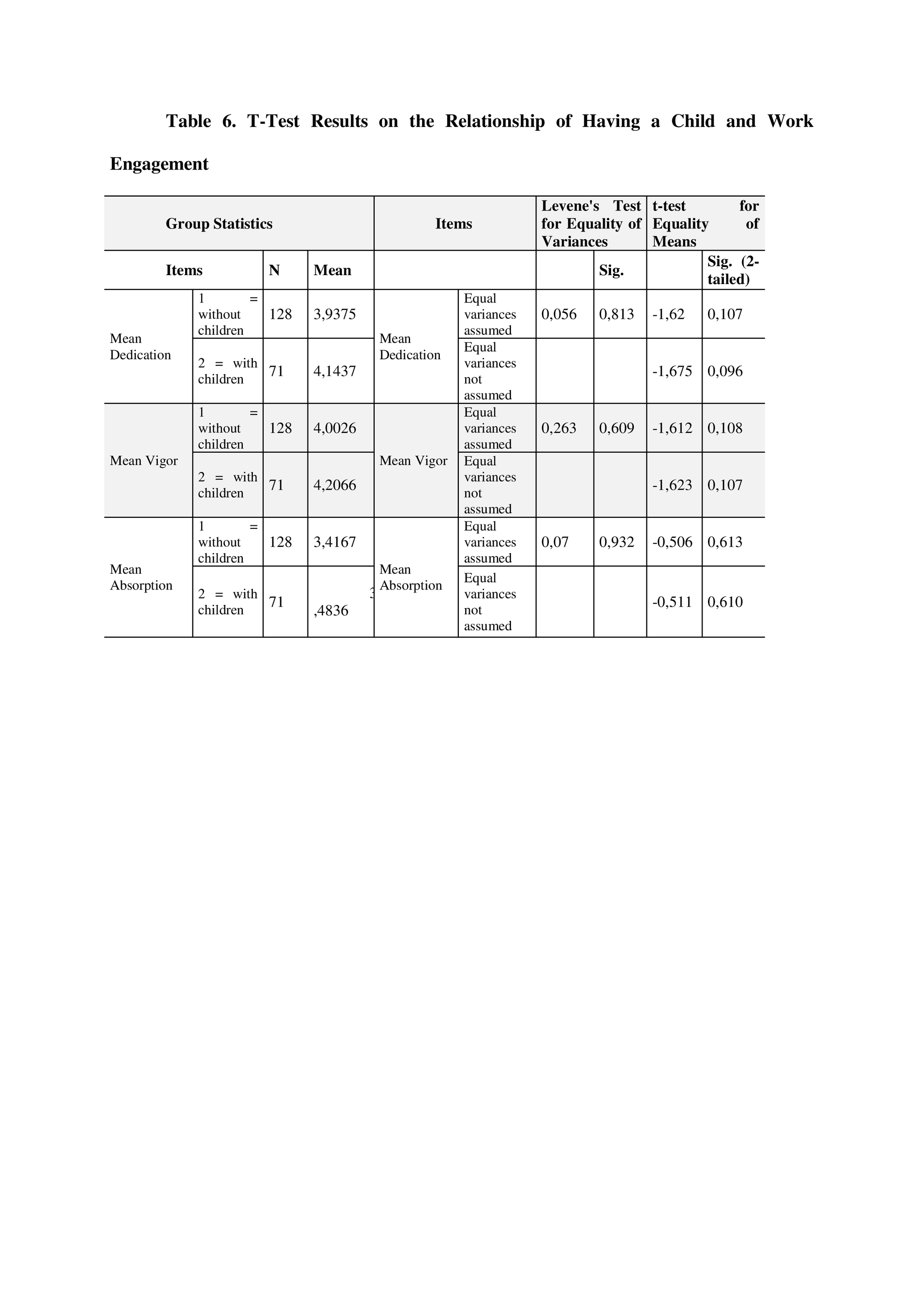

Supplement: S6 Table — (TIF) [file pone.0276784.s007.tif]
