# Supplementary material for: The relationship of work engagement with job experience, marital status and having children among flexible workers after the Covid-19 pandemic
Source: PLoS One. 2022 Nov 11;17(11):e0276784. doi: 10.1371/journal.pone.0276784 (PMC9651564; doi:10.1371/journal.pone.0276784)
Supplement: S1 Appendix — (DOCX) [file pone.0276784.s008.docx]

**APPENDIX I**

| **Work Engagement Questionnaire** |
| --- |
| İşimi yaparken enerji dolu olurum. |
| İşimde kendimi güçlü ve dinç hissederim. |
| İşimde zihinsel olarak oldukça dayanıklıyım. |
| Sabah uyandığımda işe gitmek için istekli olurum. |
| Çok uzun saatler çalışabilirim. |
| Her şey yolunda gitmese bile işimde daima sebat ederim. |
| Çalışırken işime dalıp giderim. |
| Çalışırken yaptığım işe kendimi kaptırırım. |
| Çalışırken çevremdeki her şeyi unutuveririm. |
| Çalışırken mola vermekte zorlanırım. |
| Çalışırken zamanın nasıl geçtiğini anlamam. |
| Yoğun çalıştığım zamanlarda kendimi mutlu hissederim. |
| Yaptığım işin anlamlı olduğunu ve bir amaca hizmet ettiğini düşünüyorum. |
| İşimin ilgi çekici ve gayret gerektiren bir iş olduğunu düşünüyorum |
| Yaptığım işle gurur duyarım. |
| İşim bana ilham verir. |
| İşimi hevesle yaparım. |
