# Supplementary material for: The relationship of work engagement with job experience, marital status and having children among flexible workers after the Covid-19 pandemic
Source: PLoS One. 2022 Nov 11;17(11):e0276784. doi: 10.1371/journal.pone.0276784 (PMC9651564; doi:10.1371/journal.pone.0276784)
Supplement: Attachment — Submitted filename: GENERAL COMMENTS FROM REVIEWER.pdf [file pone.0276784.s010.pdf]

GENERAL COMMENTS FROM REVIEWER

MANUSCRIPT NUMBER: PHONE-D-22-08087

I. The abstract is not clear, re-write it considering context, aim(s), method, results and implications.
II. Novelty and contribution should be clarified.
III. Motivation behind the work should be forcefully discussed.
IV. More discussions are essential.
V. Comparison analysis with more articles should be discussed.
