# Supplementary material for: The relationship of work engagement with job experience, marital status and having children among flexible workers after the Covid-19 pandemic
Source: PLoS One. 2022 Nov 11;17(11):e0276784. doi: 10.1371/journal.pone.0276784 (PMC9651564; doi:10.1371/journal.pone.0276784)
Supplement: Attachment — Submitted filename: PONE-D-22-08087_reviewer.docx [file pone.0276784.s009.docx]

PLOS ONE

The Relationship of Work Engagement with Job Experience, Marital Status and Having

Children Among Flexible Workers After the Covid-19 Pandemic

--Manuscript Draft--

| Manuscript Number: | PONE-D-22-08087 |
| --- | --- |
| Article Type: | Research Article |
| Full Title: | The Relationship of Work Engagement with Job Experience, Marital Status and Having Children Among Flexible Workers After the Covid-19 Pandemic |
| Short Title: | The Relationship of Work Engagement with Job Experience Among Flexible Workers After the Covid-19 Pandemic |
| Corresponding Author: | Adnan Veysel Ertemel, PhD  Istanbul Commerce University: Istanbul Ticaret Universitesi istanbul, Istanbul TURKEY |
| Keywords: | flexible work arrangements; COVID-19 pandemic; work engagement; job experience; marital status; children |
| Abstract: | The COVID-19 pandemic has brought about serious consequences in business world practices. Among these, flexible working policies have increased to a great extent. This has resulted in serious problems in the work-life balance. In this context, conditions such as having children and marital status have been important factors that can affect work engagement among flexible workers in the post- COVID era. Therefore, this study investigates the effect of marital status, job experience and having children on work engagement among white-collar workers who work in flexible hours. The research is conducted on 199 employees. The results indicate that the level of absorption of the employees changes according to their marital status, and yet, the work engagement is not related to having children. In addition, it is seen that there is a significant relationship between job experience and work engagement. |
| Order of Authors: | Murat Çemberci |
|  | Mustafa Emre Civelek, Associate Professor |
|  | Adnan Veysel Ertemel, PhD |
|  | Perlin Naz Cömert |
| Additional Information: |  |
| Question | Response |
| Financial Disclosure  Enter a financial disclosure statement that describes the sources of funding for the work included in this submission. Review the [submission guidelines](http://journals.plos.org/plosone/s/submission-guidelines#loc-financial-disclosure-statement) for detailed requirements. View published research articles from [PLOS ONE](http://journals.plos.org/plosone/) for specific examples.  This statement is required for submission and will appear in the published article if the submission is accepted. Please make sure it is accurate. | The author(s) received no specific funding for this work. |

| Unfunded studies Enter: The author(s) received no specific funding for this work. Funded studies Enter a statement with the following details: - Initials of the authors who received each award - Grant numbers awarded to each author - The full name of each funder - URL of each funder website - Did the sponsors or funders play any role in the study design, data collection and analysis, decision to publish, or preparation of the manuscript? - NO - Include this sentence at the end of your statement: The funders had no role in study design, data collection and analysis, decision to publish, or preparation of the manuscript. - YES - Specify the role(s) played. |  |
| --- | --- |
| * typeset |  |
| Competing Interests  Use the instructions below to enter a competing interest statement for this submission. On behalf of all authors, disclose any [competing interests](http://journals.plos.org/plosone/s/competing-interests) that could be perceived to bias this work—acknowledging all financial support and any other relevant financial or nonfinancial competing interests.  This statement is required for submission and will appear in the published article if the submission is accepted. Please make sure it is accurate and that any funding sources listed in your Funding Information later in the submission form are also declared in your Financial Disclosure statement.  View published research articles from [PLOS ONE](http://journals.plos.org/plosone/) for specific examples. | The authors have declared that no competing interests exist. |

| NO authors have competing interests Enter: The authors have declared that no competing interests exist. Authors with competing interests Enter competing interest details beginning with this statement: I have read the journal's policy and the authors of this manuscript have the following competing interests: [insert competing interests here] |  |
| --- | --- |
| * typeset |  |
| Ethics Statement  Enter an ethics statement for this submission. This statement is required if the study involved:   - Human participants - Human specimens or tissue - Vertebrate animals or cephalopods - Vertebrate embryos or tissues - Field research   Write "N/A" if the submission does not require an ethics statement.  General guidance is provided below. Consult the [submission guidelines](http://journals.plos.org/plosone/s/submission-guidelines#loc-guidelines-for-specific-study-types) for detailed instructions. Make sure that all information entered here is included in the Methods section of the manuscript. | N/A |

Format for specific study types

Human Subject Research (involving human

participants and/or tissue)

Give the name of the institutional review

board or ethics committee that approved the

study

•

Include the approval number and/or a

statement indicating approval of this

research

•

Indicate the form of consent obtained

(

written/oral) or the reason that consent was

not obtained (e.g. the data were analyzed

anonymously)

•

Animal Research (involving vertebrate

animals, embryos or tissues)

Provide the name of the Institutional Animal

Care and Use Committee (IACUC) or other

relevant ethics board that reviewed the

study protocol, and indicate whether they

approved this research or granted a formal

waiver of ethical approval

•

Include an approval number if one was

obtained

•

If the study involved

non-human primates

,

add

additional details

about animal welfare

and steps taken to ameliorate suffering

•

If anesthesia, euthanasia, or any kind of

animal sacrifice is part of the study, include

briefly which substances and/or methods

were applied

•

Field Research

Include the following details if this study

involves the collection of plant, animal, or

other materials from a natural setting:

Field permit number

•

Name of the institution or relevant body that

granted permission

•

Data Availability

Authors are required to make all data

underlying the findings described fully

available, without restriction, and from the

time of publication. PLOS allows rare

exceptions to address legal and ethical

concerns. See the

[PLOS Data Polic](http://journals.plos.org/plosone/s/data-availability)

[y](http://journals.plos.org/plosone/s/data-availability)

and

[FA](http://journals.plos.org/plosone/s/data-availability#loc-faqs-for-data-policy)

[Q](http://journals.plos.org/plosone/s/data-availability#loc-faqs-for-data-policy)

for detailed information.

Yes - all data are fully available without restriction

| A Data Availability Statement describing where the data can be found is required at submission. Your answers to this question constitute the Data Availability Statement and will be published in the article, if accepted. |  |
| --- | --- |
| Important: Stating ‘data available on request from the author’ is not sufficient. If your data are only available upon request, select ‘No’ for the first question and explain your exceptional situation in the text box. |  |
| Do the authors confirm that all data underlying the findings described in their manuscript are fully available without restriction? |  |
| Describe where the data may be found in full sentences. If you are copying our sample text, replace any instances of XXX with the appropriate details.   - If the data are held or will be held in a public repository, include URLs, accession numbers or DOIs. If this information will only be available after acceptance, indicate this by ticking the box below. For example: All XXX files   are available from the XXX database (accession number(s) XXX, XXX.).   - If the data are all contained within the manuscript and/or Supporting Information files, enter the following: All relevant data are within the manuscript and its Supporting Information files. - If neither of these applies but you are able to provide details of access elsewhere, with or without limitations, please do so. For example:   Data cannot be shared publicly because of [XXX]. Data are available from the XXX Institutional Data Access / Ethics Committee (contact via XXX) for researchers who meet the criteria for access to confidential data.  The data underlying the results presented in the study are available from (include the name of the third party | https://figshare.com/s/631d0c6cb9299ea95bdc |

| and contact information or URL).  • This text is appropriate if the data are owned by a third party and authors do not have permission to share the data.  * typeset |  |
| --- | --- |
| Additional data availability information: |  |

[Click here to access/download;Manuscript;The Relationship of Employee Passion w v.7.docx](https://www.editorialmanager.com/pone/download.aspx?id=30838125&guid=ada89825-2106-4899-a9f5-b60340c898b3&scheme=1)

**The Relationship of Work Engagement with Job Experience, Marital**

**Status and Having Children Among Flexible Workers After the Covid-19**

# Pandemic

**Murat Çemberci ^1^, Mustafa Emre Civelek ^2^ Adnan Veysel Ertemel ^3^* Perlin Naz Cömert**

**4**

1 Business Administration, Yıldız Technical University, Istanbul, Turkey
2 Business Administration, Istanbul Commerce University, Istanbul, Turkey
3 Business Administration, Istanbul Commerce University, Istanbul, Turkey *
4 Business Administration, Yıldız Technical University, Istanbul, Turkey

*****	Corresponding Author: avertemel@ticaret.edu.tr

# Abstract

The COVID-19 pandemic has brought about serious consequences in business world practices. Among these, flexible working policies have increased to a great extent. This has resulted in serious problems in the work-life balance. In this context, conditions such as having children and marital status have been important factors that can affect work engagement among flexible workers in the post- COVID era. Therefore, this study investigates the effect of marital status, job experience and having children on work engagement among white-collar workers who work in flexible hours. The research is conducted on 199 employees. The results indicate that the level of absorption of the employees changes according to their marital status, and yet, the work engagement is not related to having children. In addition, it is seen that there is a significant relationship between job experience and work engagement.

**Keywords:** flexible work arrangements, COVID-19 pandemic, work engagement, job

experience, marital status, children

# Introduction

In the 21st century work environment, most employees need to decide on their own work schedules and workplaces in order to adapt to more individualized work times and family constraints. Therefore, flexible work arrangements can become a solution to reduce the problems of employees by helping them about maintaining a balance between work life and family life [1].

Although the prevalence of flexible working arrangements has increased sharply over the past decade, there has been a dramatic increase in this trend after the outbreak of the COVID-19 pandemic [2]. World Health Organization’s (WHO) declaration of the COVID-19 outbreak as an international pandemic on March 11, 2020, have resulted in quarantine and “work from home” practices in many countries [3]. Thus, the COVID -19 epidemic caused a radical change in the business world in a short time. Actually, along with closures and transitions to new business regulations, the pandemic has created a significant impact on society and the global economy at large. Quarantine has forced most office workers to work from home and organizations to adapt quickly to the new situation with technological and managerial support and adopt new working practices [4]. During this period, the conflict between work and family responsibilities for families with children has increased. In particular, the work-life balance for many workers has been affected seriously as the boundaries between the responsibilities of work and family life have been blurred [5]. At the same time, COVID 19 has increased the burden on women. Since the pandemic began, 60% of women and 54% of men have stated that their domestic work has increased [6]. Specifically, the pandemic has increased the need for data-driven, short-term, yet sustainable solutions to many problems that arise as a result of a diverse and multi-generational workforce mix with different expectations regarding flexible work arrangements [5]. In their study, Taveras et al. [3] has found out that getting used to working from home is regarded as easy for employees. The same study also indicates that lack of interaction with coworkers, lack of resources related to support infrastructures such as the internet or printer, and compensating for remote work with housework and childcare were highlighted among the main difficulties encountered. Transformations in the world and the working environment require further exploration of flexible work arrangements and examining the extent to which flexible work arrangements can help organizations achieve desired results. Accordingly, flexible working arrangements such as working flexible hours, job sharing and working from home are attracting the attention of organizations. In essence, flexible work arrangements aim at improving employee wellbeing, work-life balance and firm performance [7].

In 2020, about 56% of workers in the United States had a job that could be done from home at least partially because of the fact that their job was knowledge-based and had no physical work requirements [8]. In a study conducted in 2021, it was seen that 50% of the contract workers preferred to work independently because of the freedom and flexibility it provides [9]. Also, the majority of millennials who work say they prefer flexible work arrangements, including the ability to work from home [10]. According to the results of the 'The Impact of the Coronavirus Outbreak on Business Life Survey' conducted in Turkey with the participation of 167 companies, 103 of which are global and 64 of which are local, the rate of companies that had the practice of working from home before the coronavirus was 45 percent, while it has reached to 95 percent for the head office em-ployees [11]. More than 40,7 percent of the companies had stated that they had difficulties in employee motivation during the pandemic. Companies have accelerated their infra-structural actions to adapt to new working practices and they still continue to take the necessary steps for the adaptation of their employees. More than 72 percent of companies’ state that they encourage their employees to use

digital solutions in order to accelerate adaptation to remote working [11].

Various studies in the extant literature emphasize the advantages of flexible working, such as increasing productivity, work satisfaction, work-life balance, commitment, reducing absenteeism and work-life conflict [5, 12, 13, 14]. A study conducted with 232 working adults showed that the availability of flexible time and flexible location lead to higher work engagement and organizational commitment [15]. Similarly, Weidemen and Hofmeyr [16] found a positive relationship between flexible working arrangements and work engagement. The study undertaken by Richman et al. [17] also showed that workplace flexibility can increase work engagement. Likewise, Zaman and Ansari [18], who has found a positive relationship between workplace flexibility and work engage-ment underline that flexible business alternatives are an important factor affecting work engagement in the IT industry. Sonnentag et al. [19] observed that being psychologically disconnected from work during non-working times is a key factor in the formation of high levels of work engagement. According to Pocock's [20] study, one-third of the employees need but do not demand flexibility in order to achieve better harmony and balance in their work and non-work lives. Some studies have shown that flexible working arrangements are associated with better employee well-being [21, 22, 23], higher productivity and performance [12, 24, 25, 7] In addition to its advantages, flexible and remote working could also lead to some negative consequences. For example, it can be said that working remotely during the pandemic period reduces the effects on work-life balance and increases work-family conflict [5]. According to Palumbo's [26] research, working from home has caused public sector workers to be exposed to increased work-life conflict. In the same study, it was observed that working from home increased work-related fatigue and worsened the perception of work-life balance [26]. In addition, it is seen that flexible working has some disadvantages such as difficulties in career management and performance evaluation as well as difficulty in participating in social activities. Moreover, despite past research and theories emphasizing the relationship between flexibility and work engagement, Timms et al. [27] found a negative relationship between the use of flexible work arrangements and work engagement. As a consequence, it is observed that there are different research results on the effects of flexible working on employees and organizational results. In this context, it seems that past research results with conflicting results and the changes caused by the COVID-19 epidemic underline the need for further research on this issue.

In addition, there is a need for more research on how work engagement, which is an important variable found to be associated with performance, organizational citizenship behavior and customer satisfaction, changes with the spread of flexible working ar-rangements in the post-COVID-19 era [28]. Specifically, demographic characteristics of the employees are examined with regard to work engagement in this study. This is due to the fact that the personal obligations of the employees and the conflict of their work lives are felt more intensively in the pandemic period. In this context, it is assumed that the level of work engagement among flexible workers will change according to having children, marital status and experience. Although some previous studies show the relationship between demographic variables and work engagement, some other studies show the opposite. Hence, this relationship needs to be elaborated further in the post-COVID-19 period. Having a child and marital status stand out as important factors that may be re-lated to work engagement, especially in the postCOVID era. Smith and Dumas [29], Fukuzaki et al. [30], Poulsen et al. [31] found that the number of children is a factor that has an impact on work engagement. Based on the previous studies, it is assumed that there is a significant relationship between work engagement and having a child in this study. Likewise, the extant literature demonstrates that work engagement will vary ac-cording to marital status and job experience [32, 33, 34]. Based on the previous studies, this study assumes that there is a relationship between marital status and work engage-ment in flexible workers. Most past studies show that there is a relationship between job experience and work engagement [35, 36, 37]. For this reason, the relationship between job experience and work engagement is also investigated in this study. There is a necessity of reexamining the relationships between the aforementioned variables in the new working environment. In addition, conflicting results in similar studies conducted in the past on this subject underline the need for further research in this area.

Social exchange and commitment-trust theories constitute the theoretical basis of the study. Social exchange theory states that employees are ready to offer their labor and loyalty in return for the economic and social benefits offered by the organization in the employee-employer relationship [38]. Social exchange theory proposes that flexible work arrangements will reciprocate the employer's effort by generating a sense of gratitude in employees, demonstrating higher motivation and loyalty for their work [39]. The com-mitment-trust theory argues that when commitment and trust exist together, there will be increased efficiency, productivity and efficiency in the work environment [38]. In this context, these theories are used as the baseline while investigating whether the engage-ment of employees who use flexible working options in marital status, level of job expe-rience and whether they have children have changed. The study consists of six parts. After the introduction, a literature review on the conceptual background of the research variables is presented. In the third part, the related concepts are examined with the support of the previous literature and hypotheses are developed accordingly. The fourth section describes the research methodology. In the fifth section, discussion and implica-tions, the results are outlined in relation to the previous research and how research findings can contribute to managers is explained. Finally, the limitations of the research are mentioned in the last part.

# Conceptual Background

## Flexible Work Options

The number of employees in flexible employment options has been on the rise in recent years. Flexible working is defined as the ability to decide where, when and how to work [40]. Among flexible working types are flextime, in which employees determine the start and end time of work, compressed work week, which includes employees working four long days instead of working five regular days, telecommuting, which refers to em-ployees working from home by making use of information technologies, and part-time work [27]. Spreitzer et al. [41] divided flexible work arrangements into three groups, which are flexibility in work schedule (the ability of the employee to set their own working hours, including compressed work weeks, non-standard working hours such as nights and weekends), flexibility in the location of the work (such as working from home), and flexibility in employment relationships (such as short-term contract workers, freelancers). According to a study conducted on employees participating in flexible working at an IBM company in the USA, 74% of the employees stated that they would leave IBM if it were not for flexible working, and 59% stated that they would leave IBM to find a more flexible work [42]. Abbott et al., [43] found that ignoring the commitment and responsibilities of employees in their personal lives at work has costly organizational consequences such as high absenteeism and increased staff turnover. Accordingly, flexible working arrangements are designed to balance the conflicting personal and work demands of employees [44]. In this context, practices that improve employees' work-life balance are considered to be important since they will also be beneficial to organizations. Flexible working is recommended as a family-friendly policy that allows balancing work-life responsibilities [42].

There are several advantages and disadvantages to adopting flexible working. In-creasing the ability to recruit and retain employees is one of these advantages [5]. New forms of work, such as flexible working, have been shown to increase work engagement and work satisfaction and reduce turnover [40, 17, 45]. Kelliher and Anderson's study [46] showed that there is a strong and positive relationship between flexible working and perceived work quality. In addition, informal teleworking arrangements have been found to have a positive indirect effect on employee performance through organizational commitment and work satisfaction [47]. In addition, it has been observed that flexible working time increases employees' autonomy and feedback perception, increasing productivity and work satisfaction and reducing absenteeism [12]. It has been observed that flexible work arrangements reduce employee stress and increase work-life balance [5]. Similarly, according to Golden et al. [13] study, remote working reduces work-life conflict by enabling employees to better meet family needs. The increase in employees' control over their work schedules leads to organizational support, increased perception of work-family balance and organizational commitment [14, 48] Flexible working has also enabled older adults who do not want to work full time but have valuable knowledge and job experience to offer to organizations [49]. It has been observed that flexible working increases work engagement and reduces work-family conflict [50]. According to Carless and Wintle [51] , flexible solutions such as flexible hours and remote working make organizations more attractive to potential employees. Flexibility has been shown to reduce employee turnover and absenteeism and also to increase productivity [52]. It has been observed that women working in companies that offer flexible work options have higher work satisfaction, lower absenteeism, work more in their own time and work until later in their pregnancy [53]. Scandura and Lankau [54] suggested that as a result of providing flexible work arrangements to employees, employees feel the need to respond to this situation with higher commitment, loyalty and work performance mentioning the positive results of the psychological contract between the employer and the employee. This is particularly common in women. According to commitment and trust theory, when commitment and trust exist together, there is an increase in productivity, productivity and efficiency. Therefore, policies that help reduce the problems of employees and provide work-life balance will increase the employee’s commitment to the organization, and the relationship of loyalty and trust between the employee and the employer will also increase. This will also affect organizational performance in a positive way. In a past study, employee satisfaction and work-family balance improved when employers provided flexibility of work schedules [1]. It is claimed that working from home as a flexible working schedule increases employees' control over the spatio-temporal work context and improves the quality of organizational activities [55]. On the other hand, Shifrin and Michel [2] demonstrate that flexible work arrangements result in better physical health and reduced absenteeism, which underlines the positive impact of flexible working on the health of workers. A previous study found that flexible time workers have higher work satisfaction [56]. It has been seen that flexible working policies will increase the wellbeing of employees by providing higher temporal flexibility [57].

In contrast to the positive results listed above, Timms et al.'s study [27] found a negative relationship between work engagement and flexible work arrangements. Although organizations aim to facilitate the work of employees by providing flexible working arrangements, some studies suggest that managers could see the need for flexibility in employees as a sign of decreasing work engagement [44, 58] In this context, employees are hesitant to take advantage of the convenience of flexible working, considering that flexible working policies may result in career barriers. Accordingly, employees think that being physically present at the workplace can better reflect their commitment to work in the eyes of managers compared to flexible working [57]. In addition to these, flexible working could bring about difficulties in managing career development and professional image, declining income, difficulty in participating in social activities at work and professional networking. In addition, some managerial barriers may be encountered in flexible working such as work coordination and scheduling problems, difficulties in evaluating performance and equality issues. As a consequence, both positive and negative effects of flexible working are encountered in the extant literature. This stems from conflicting research results on this subject. At the same time, it has been observed that the attitude towards flexible working options varies depending on gender, sector and previous

participation in flexible working [42].

## Work Engagement

Work engagement refers to a positive, emotional-motivational high energy state with a high level of dedication and strong focus on work [59]. It has been shown that work engagement can improve employee performance, health and well-being [60]. Work engagement is defined as a positive work-related mental state characterized by three dimensions: vigor, dedication, and absorption [61]. According to Salanova & Schaufeli [62], work engagement is an indicator of the employees' intrinsic motivation. It has also been found that work engagement is associated with a high level of creativity, task per-formance, organizational citizenship behavior and customer satisfaction [63]. Accord-ingly, individuals who are engaged about work are highly energetic, enthusiastic and fully immersed in their business activities. Employees who are engaged find the work energizing and feel a personal satisfaction about the work they do [27]. Understanding the antecedents and consequences of work engagement is important for organizations because lack of work engagement is costly [64].

Vigor is the first dimension of work engagement, which expresses the feeling of high energy and mental resilience while working, putting effort into one's work and being persistent in the face of difficulties in the work. The second dimension of work en-gagement, which is dedication refers to a strong involvement in one's own work and feeling of meaning, enthusiasm, pride, inspiration and struggle related to one's work. The third dimension is absorption which means that the individual concentrates completely on his work, feels that time passes quickly while doing work, and has difficulty in dis-connecting from work [65]. In short, vigor indicates endurance as well as the employee's motivation to exert effort; dedication reflects a strong identification with one's job, as it expresses work engagement and enthusiasm for work, whereas absorption refers to the focus and immersion of employees in the work [66]. According to Kahn [67], employees are more engaged in work situations that offer them more psychological meaning and confidence, and when they are psychologically available, employees tend to me more engaged. The past studies have shown that social support, performance feedback, skill variety, autonomy and learning opportunities from managers and colleagues are posi-tively associated with work engagement [63]. In addition, the extant literature indicates that work engagement is strongly related with financial returns, work performance, in-tention to leave and employee creativity [68, 69, 70, 71]. Among the factors that lead to work engagement, one of the most interesting factors is job resources. It refers to physical, social or organizational resources that contribute to reducing business costs, achieving business goals and individual development. Social support, work control, participation in decision making, feedback, rewards, work security and task diversity can be classified as some of the business resource types. According to previous studies, social support, performance feedback and executive coaching were found to be associated with work engagement [70]. Another study has discovered that job resources such as employee empowerment, managerial support, feedback and development opportunities, and individual resources such as self-efficacy and optimism are among the predictors of work engagement

[72]. The concept of work engagement was first theorized by Kahn [67] and then operationalized by Maslach et al. [73]. The present study was guided by Schaufeli and Bakker's conceptualization of work engagement.

# Development of Hypotheses

## The Relationship Between Having Children and Work Engagement

Although various past studies have found a relationship between having children and the number of children and work engagement, it is important to reexamine this re-lationship in the post COVID world [29, 30, 31]. According to a recent study by Del Boca et al. [74], it has been observed that women have been affected more in terms of increased housework and childcare during the COVID-19 period. The same study indicated child-care work was shared more equally among partners compared to housework. The study of Del Boca et al. [74] showed that working women with children aged 0-5 have more difficulty in maintaining work-life balance during the COVID-19 period. In another study, the time of work, marriage, child development and family stress have been found to be important [1]. Studies have shown that these flexible working arrangements allow mothers to maintain work hours and income after giving birth [75]. The study of Rajesh and Ekambaram [76], in IT sector, has shown that pregnancy, childbirth, marriage and childcare are among the most significant barriers in terms of career development. In the same study, flexible working arrangements were found to be an important facilitator for women to continue in their careers. At the same time, flexible working has also been shown to have beneficial effects on the mental health and well-being of fathers and chil-dren as well as improvements in family and couple relationships [55]. In contrast, a bio-logical study of eleven key indicators of chronic stress levels in the UK found that working mothers of two children were 40% more stressed than the average person in pre-pandemic conditions [77]. In another study, it was found that young individuals have more difficulty in breaking away from work psychologically, but women with children may not have that much difficulty in breaking away from work because they are more involved in housework and child care [78]. In the study of Smith and Dumas [29] , single and childless workers were found to be less engaged than those with a family. According to a study conducted among nurses, it was observed that the children of nurses under the age of 6 had a beneficial effect on mental health [79]. It has been observed that living with children has a moderator effect that weakens the relationship between employees' positive work thoughts and their work engagement [80]. In addition, it has been observed that women job experiences more workload, stress and conflict than men, and this increases significantly with the increase in the number of children [81]. In the study of Ten Brummelhuis and Bakker [82], it was found that housework reduces vigor because it reduces relaxation. Therefore, these activities were found to significantly affect the next day's work engagement. According to the study of Fukuzaki et al. [30], there is a significant relationship between the number of children and work engagement. In the research conducted on health workers to investigate the issue of work engagement, employee and manager support, it has been found that having 16 years of or longer job experience, being directly involved in patient care, having children and not working in shifts are positively related to work engagement. In addition, high levels of work en-gagement were reported by therapists in conditions of low psychological disconnection from work, high income satisfaction, graduate qualifications, high frequency of laughing and having children [83]. According to the study of Chung and Van der Horst [84] , flexible working arrangements enable women to continue working after the birth of the first child. It has been observed that women who use flexible working hours are less likely to reduce their working hours after childbirth [84]. Therefore, it has been discovered that flexible working is a tool that improves and preserves the working capacity of individuals in the face of increasing family demands [84]. According to the suggestion of Golden [85], it is suggested that married employees and employees with young children participate in more flexible work. In this study, it is assumed that there is a significant relationship between having children and work engagement among flexible workers, with the fol-lowing hypothesis based on the previous studies.

H1a: There is relationship between having children and dedication among flexible workers

H1b: There is relationship between having children and absorption among flexible workers

H1c: There is relationship between having children and vigor among flexible workers

## The Relationship between Marital Status and Work Engagement

In a study conducted by Sharma et al. [86], it was discovered that there was no dif-ference in work engagement in terms of gender, marital status and income. Another study undertaken by Shukla, Adhikari and Singh [34], has found a significant difference in the levels of work engagement according to gender, marital status and experience. It has been observed that the level of work engagement is higher in married workers. In another study, marital status was found to have a significant and independent effect on nurses' dedication and total work engagement [33]. This can be explained by the fact that married nurses have more emotional stability than single nurses. Chan et al. [32] study revealed that age, marital status and education level affect work engagement. On the other hand, according to Kose's [87] study, marital status, branch and educational status are not significant factors for teachers' work engagement. Similarly, in Wilmer, Westerberg Jacobson, and Lindberg's [88] study, no significant relationship was found between education, marital status, age and work engagement. In addition, Othman and Nasurdin [89] argue that age, marital status and education status are not related to work engage-ment. Conflicting results in the past research and the changes brought about by the COVID-19 pandemic in the business world highlight the importance of reexamining the relationship between marital status and work engagement among flexible workers in the postpandemic period. In this context, it is assumed that there is a relationship between marital status and work engagement.

H2a: There is relationship between marital status and dedication among flexible workers

H2b: There is relationship between marital status and absorption among flexible workers

H2c: There is relationship between marital status and vigor among flexible workers

## The Relationship between Job Experience and Work Engagement

Some scholars argue that there is an increase in work engagement as employees become more experienced [35, 36]. However, Mayer and Schoorman [90] observed the opposite indicating that there is a statistically significant negative relationship between job experience and work engagement. According to a study conducted by Faskhodi and Siyyari [37], among teachers work engagement increases as years of experience increase. In the study of Shukla et al. [34], it was seen that there was a significant difference in levels of work engagement depending on job experience. According to the study of Sharma et al. [86], it was found that there is a significant positive relationship between work engagement and age, education level and job experience. In the study of Bamford et al. (2013), the number of years of nursing job experience was found to be significantly and positively related to work engagement [91]. Poulsen et al. [31] showed that there is a positive relationship between job experience and work engagement. In the study of Bell and Barkhuizen [92], it was seen that there was no difference in work engagement in groupings according to gender, organizational level, number of years in the position and years of job experience. As can be seen, there are different results in the literature on the relationship between job experience and work engagement. This indicates that more re-search is needed on this subject. As has been discovered many times in the past literature, it is assumed in this study that there is a significant relationship between job experience and work engagement. In this context, the hypothesis developed with support from previous studies is as follows:

H3a: There is relationship between job experience and dedication among flexible workers

H3b: There is relationship between job experience and absorption among flexible workers

H3c: There is relationship between job experience and vigor among flexible workers

**Figure 1. Research Model**

# Materials and methods

Quantitative research method was used in this study. Both scales used are 5-dimensional Likert scales. Expressions on the Likert scale range from 1-Strongly Disagree and 5-Strongly Agree. In order to measure the validity of the scales, factor analysis was per-formed with the help of SPSS Statistics program. Then, Cronbach's alpha values were calculated to determine the reliability of the scales. Finally, correlation analysis, T-test and ANOVA test were performed to test the hypotheses.

## Measures and Sampling

To measure work engagement, the sub-dimensions measured are as follows: Vigor, Dedication and Absorption. The 17-item Utrecht Work Engagement Scale was used to measure the

aforementioned constructs [63, 93]. Six of the items in the scale measure vigor, five items measure dedication and six items measure absorption. All the statements in the questionnaire were rated on a 5point Likert scale where participants indicated whether they agreed with each statement (1 = Strongly Disagree, 5 = Strongly Disagree). 199 valid questionnaires were collected from flexible workers. The sample of the study consists of flexible workers. Convenience sampling method with voluntary response was used. The scale used is given in the appendix of the study.

## Construct Validity and Reliability

In order to establish the dimensionality of the scale, a principal factor analysis with varimax rotation was conducted on the original 17 items of Utrecht Work Engagement Scale. As a result of the factor analysis, questions 4, 5, 6, 8 and 11 were removed from the work engagement scale. 3 questions were removed from the vigor scale and 3 questions from the absorption scale. Thus, the analysis continued with the 11 questions on the work engagement scale. As a result of the Cronbach's Alpha reliability test performed after the factor analysis, the Cronbach's Alpha reliability coefficient was 0.888 for vigor scale, 0.919 for dedication scale and 0,7 for absorption scale. The three dimensions and factor load in the work engagement scale are shown in Table 1.

**Table 1. Factor and Reliability Analysis Results**

| **Items** |  | **Factor loads** | | **Alpha** | **Cronbach** |
| --- | --- | --- | --- | --- | --- |
| Dedication3 | ,887 | 0 |  |  |  |
| Dedication4  Dedication5 | ,861  ,851 | 0  0 |  |  | 0,919 |
| Dedication2 | ,816 | 0 |  |  |  |
| Dedication1 | ,793 | 0 |  |  |  |
| Vigor2 |  | ,920 | 0 |  |  |
| Vigor1 |  | ,903 | 0 |  | 0,888 |
| Vigor3 |  | ,810 | 0 |  |  |
| Absorption3 |  |  | ,841 | 0 |  |
| Absorption4 |  |  | ,758 | 0 | 0,700 |
| Absorption1 |  |  | ,734 | 0 |  |

366 *Extraction Method: Principal Component Analysis.*
367 *Rotation Method: Varimax with Kaiser Normalization.*
368 *a. Rotation converged in 5 iterations.*

# Results

T-test was conducted to test the relationship between work engagement and marital status. According to the results, there is a difference in absorption, which is the sub-dimension of work engagement, according to marital status.

**Table 2. T-Test Results on the Relationship between Marital Status and Work Engagement**

| **Group Statistics** | | |  | **Items** | | **Lev ene's Test for Equality of**  **Variances** | | **T-**  **test for**  **Equality of**  **Means** | |
| --- | --- | --- | --- | --- | --- | --- | --- | --- | --- |
| **It ems** | **arital**  **Status** | **M** | **ean** |  | |  | **ig.** |  | **ig. (2tailed)** |
| M  ean Dedication | arried | M  12 | ,0357 | M  ean Dedication | E  qual variances assumed | ,012 | ,316 | ,456 | ,649 |
|  | ingle | S  7 | ,9793 |  | E  qual variances not assumed |  |  | ,447 | ,656 |
| M ean Vigor | arried | M  12 | ,1250 | M ean Vigor | E  qual variances assumed | ,485 | ,117 | ,925 | ,356 |
|  | ingle | S  7 | ,0115 |  | E  qual variances  not assumed |  |  | ,948 | ,345 |
| M  ean Absorption | arried | M  12 | ,5625 | M  ean Absorption | E  qual variances assumed | ,084 | ,772 | ,21 | ,028 |
|  | ingle | S  7 | ,2835 |  | E  qual variances  not assumed |  |  | ,20 | ,029 |

The t-test results are presented in Table 3. According to this result, it is seen that the level of absorption of the employees change depending on whether they are married or not. It can be interpreted that married people are more concentrated on their work because their job experience increases in direct proportion to the possibility of being older than singles and because they stay at the place they work for a long time.

**Table 3. ANOVA Test Results on the Relationship between Job Experience and Work Engagement**

| **ANOVA** | |  |  | | | **Test of Homogeneity of Variances** | | | |
| --- | --- | --- | --- | --- | --- | --- | --- | --- | --- |
| **Items** | **Group**  **s** |  | **Mean** | **Sig.** | | **Items** | | **Levene**  **Statisti c** | **Sig.** |
| Mean Vigor |  | 1 | 3,8889  4,0057  4,2259 | Between Groups | ,061 | Mean Vigor | Based on Mean | ,015 | ,364  ,379  ,379 |
|  |  | 8 |  |  |  |  |  | ,976 |  |
|  |  | 0 |  |  |  |  |  | ,976 |  |
| Mean  Dedication |  | 1 | ,9843 | Between Groups | ,781 | Mean Dedicati on | Based on Mean | ,483 | ,618 |
|  |  | 8 | ,9621 |  |  |  |  | ,517 | ,597 |
|  |  | 0 | ,0578 |  |  |  |  | ,517 | ,597 |
| Mean  Absorption |  | 1 | ,366  ,5172  ,4333 | Between Groups | ,676 | Mean Absorpti on | Based on Mean | ,548 | ,215  ,232  ,232 |
|  |  | 8 |  |  |  |  |  | ,47 |  |
|  |  | 0 |  |  |  |  |  | ,47 |  |

*Group 1: 0-5 years*

*Group 2: 5-10 years*

*Group 3: +10 years*

Table 4 shows that there is a significant relationship between job experience and work engagement. Participants were classified into 3 different groups in terms of their expe-rience. The first group consists of those with up to 5 years of experience, the second group consists of those with 5 to 10 years of experience, and the third group consists of those with more than 10 years of experience. It is seen that those with 5 to 10 years of job experience have the highest average in terms of absorption. Looking at the averages, it is seen that more experienced employees are more dedicated and more vigorous. It is seen that the two groups which are more experienced, have higher absorption than the group with less experience.

**Table 4. T-Test Results on the Relationship of Having a Child and Work Engagement**

| **Group Statistics** | | |  | **Items** | | **Leven e's Test for Equality of**  **Variances** | | **t-test**  **for Equality of Means** | |
| --- | --- | --- | --- | --- | --- | --- | --- | --- | --- |
| **Items** | | **N** | **Mean** |  | |  | **Sig.** |  | **Sig. (2tailed)** |
| Mean  Dedication | 1  = without children | 28 | 3,9375 | Mean  Dedication | Equal variances assumed | ,056 | ,813 | 1,62 | ,107 |
|  | 2 = with  children | 1 | 4,1437 |  | Equal variances not assumed |  |  | 1,675 | ,096 |
| Mean Vigor | 1  = without children | 28 | 4,0026 | Mean Vigor | Equal variances assumed | ,263 | ,609 | 1,612 | ,108 |
|  | 2 = with  children | 1 | 4,2066 |  | Equal variances  not assumed |  |  | 1,623 | ,107 |
| Mean  Absorption | 1  = without children | 28 | 3,4167 | Mean  Absorption | Equal variances assumed | ,07 | ,932 | 0,506 | ,613 |

0

0

0

0

0

0

2

with

=

children

1

3

,4836

Equal

variances

not

assumed

0,511

,610

T-test was also conducted to explore the relationship between having children and work engagement. According to the results of this test, it is seen that the work engagement does not differ between those who have children and those who do not. Based on this result, it is seen that work engagement and having children are not related to each other.

# Discussion and Conclusion

Research during the COVID-19 period shows that remote work has increased [11]. In this context, it can be said that the effect of new working styles such as flexible working has started to attract more attention recently. The flexible working arrangements that came with the COVID-19 epidemic have caused the increasing burden of housework and childcare on both women and men, sudden changes in the way they work and the blur-ring of the boundaries between work and family life [6]. The existing studies show that in the aftermath of the COVID-19 pandemic, women are overwhelmed by their increased domestic obligations as well as their workloads. In this context, the need to reexamine the variables of work engagement, marital status, job experience and having children, which were examined in the previous studies and which are related, under the changing social and economic conditions in the post-pandemic period. In this context, in this study, it has been investigated whether work engagement will change according to marital status, job experience and whether or not having children among flexible workers in the post-COVID-19 pandemic period.

In the past studies, the advantages of flexible working such as increasing commitment and employee well-being and reducing work-life conflict have been emphasized [5, 13, 14]. In addition, previous studies show that there is a significant relationship between flexible working and work engagement [15, 16, 18]. In addition, the presence of children at home is one of the important factors that complicates the work of employees and prevents them from focusing on work during the pandemic. For this reason, the state of having children was also examined as one of the factors affecting work engagement in this study. Previously, there are various research results showing that work engagement changes according to the number of children [29, 30, 83]. In this study, based on the previous studies, it is assumed that there is a significant relationship between having children and work engagement among flexible workers. Similarly, there are studies showing a relationship between marital status and work engagement [34, 33]. Accordingly, it is assumed that there is a significant relationship between marital status and work en-gagement among flexible workers. The relationship between job experience and work engagement has also been discovered by various researchers [35, 36]. Supporting these findings, this study hypothesizes that there is a significant relationship between job ex-perience and work engagement among flexible workers in the post-pandemic period.

The analysis results show that that there is a difference in absorption from the sub-dimensions of work engagement, depending on whether employees are married or not. In this context, it is understood that marital status among flexible workers after the pandemic is an important factor in absorption of work. Therefore, it is understood that managers should consider marital status as an important factor while trying to increase the work engagement of the employees. In addition, in parallel with previous studies, it is seen that there is a difference in work engagement according to job experience. It is seen that as the job experience increases, so does the work engagement of the people. This result is also supported by the previous studies. Based on this result, it can be interpreted that the children of more experienced and older people can be more vigorous towards their work due to their older age. It can be interpreted that the lack of vigor of those who have just started work and those with little job experience may be due to the fact that these people are open to new job opportunities and want to try different workplaces. The results surprisingly show that whether or not to have children is associated with work engagement. This may be due to the older age of the children of the employees in the sample. At the same time, these findings may indicate that employees who spend more time with their children and for their personal lives thanks to their flexible working op-tions are satisfied with this situation. Thus, it has been observed that employees balance both their personal obligations and their work well thanks to their flexible working ar-rangements, and that domestic responsibilities such as childcare are not a factor that reduces their work engagement.

The findings of this research contribute to meeting the lack and need in the literature in the post-COVID-19 period by revealing the levels of work engagement according to the different demographic characteristics of flexible employees. Thus, it is anticipated that the findings of this study will indirectly contribute to the solution of new problems in employee behavior and work-life balance in the new post-pandemic period. At the same time, it is foreseen that this research will enlighten the current situation in this field and encourage research on these issues in the future.

This research supports social exchange and commitment-trust theories. It indirectly supports the social exchange theory, which proposes that flexible working arrangements will result in greater employee gratitude and greater motivation and loyalty to their work. The results show that even if people who are given the option of flexible working do have family obligations, their work engagement does not decrease. At the same time, the results also indirectly support the commitmenttrust theory, which argues that policies that help reduce the problems of employees, provide work-life balance and that they will positively affect the loyalty and trust relations between the employeeemployer and organizational performance.

In addition to its contributions to theory, this research is also expected to be an important guide for managers in practice. Findings showing that as job experience increases, work engagement increases, managers can use flexible working arrangements to increase the work engagement of employees with more experience. It is understood that different mechanisms should be used to increase the level of work engagement of employees who have different obligations in their personal lives. This study reminds that managers should consider and take into account the roles and duties of the employees in their personal lives as well as their roles in business life. At the same time, this research reflects the situation of employees regarding the working order after the COVID-19 outbreak and informs managers about it. In addition, it is anticipated that flexible working in the workplace has gained importance in the post-COVID-19 period and will now be a tool that will be used more by managers to improve work-life balance in future research.

## Limitations and Implications for Future Research

This research has some limitations. First, the study was conducted in Turkey, and there-fore some of the results may have been based on the characteristics of Turkish culture. For this reason, it is important to carry out this research in different countries in the future and to make a cross-cultural comparison. In addition, the application of quantitative methods is another limitation of the study. Therefore, future research can enrich the literature in this field by applying qualitative research methods on flexible working and its consequences during and after COVID-19. In addition, future studies focusing on the impact of flexible working on leadership and teamwork during the COVID-19 era are encouraged. Considering the important relationship between work engagement and performance with the characteristics of colleagues and leaders, the impact of new ways of working on leadership and teams after the COVID-19 outbreak becomes important. Furthermore, the age of the children of the employees should be included as an important variable in future studies.

It is seen that new problems and new gaps continue to occur in the business and organizational behavior literature, which has undergone a great transformation after the epi-demic. In this context, it is understood that there is a need for similar studies in this field in the literature and will continue in the future. In addition, problems such as the in-creasing burden of housework and childcare and the decrease in their participation in employment after the COVID-19 pandemic are among the most important problems that come to the fore in the new post-epidemic period [6]. Moreover, women seem to be 24% more likely than men to lose their jobs completely due to the COVID pandemic [94]. In this context, future researchers are encouraged to work towards ensuring equality between men and women as well as solving these problems.

**References**

1. Golden L. Limited Access: Disparities in flexible work schedules and work-at-home. Journal of Family and Economic Issues. 2008; 29: 86-109. doi: 10.1007/s10834-007-9090-7
2. Shifrin NV, Michel JS. Flexible work arrangements and employee health: A meta-analytic review. Work & Stress. 2021; 1-26. doi: 10.1080/02678373.2021.1936287
3. Tavares F, Santos E, Diogo A. Ratten V. Teleworking in Portuguese communities during the COVID-19 pandemic. Journal of Enterprising Communities: people and places in the global economy. 2020; 23: 1750-6204. doi: 10.1108/JEC-06-2020-0113
4. Pataki-Bittó F, Kapusy K. Work environment transformation in the post COVID-19 based on work values of the future workforce. Journal of Corporate Real Estate. 2021; 23: 151-169. doi: 10.1108/JCRE-08-2020-0031
5. Waples EP, Brock-Baskin ME. Not Your Parents’ Organization? Human Resource Development Practices for Sustainable Flex Work Environments. Advances in Developing Human Resources. 2021; 23: 153-170. doi: 10.1177/1523422320982933
6. United	Nations	Women	[Internet].	Available	from	[cited	2022	Feb	28]:

[https://data.unwomen.org/publications/whose-time-care-unpaid-care-and-domestic-workhttps://data.unwomen.org/publications/whose-time-care-unpaid-care-and-domestic-work-during-covid-19during-covid-19](https://data.unwomen.org/publications/whose-time-care-unpaid-care-and-domestic-work-during-covid-19)

7. Avgoustaki A, Bessa I. Examining the link between flexible working arrangement bundles and employee work effort. Human Resource Management. 2019; 58: 431-449. doi: 10.1002/hrm.21969
8. Como R, Hambley L, Domene J. An exploration of work-life wellness and remote work during and beyond COVID-19. Canadian Journal of Career Development. 2021; 20: 46-56.
9. Bakaç C, Zyberaj J, Barela JC. Predicting employee telecommuting preferences and job outcomes amid COVID-19 pandemic: a latent profile analysis. Current Psychology. 2021; 1-

16. doi: 10.1007/s12144-021-02496-8

10. Deloitte	[Internet].	Available	from	[cited	2022	Feb	28]:

https://www2.deloitte.com/tr/tr/pages/human-capital/articles/COVID-19-sonrasi-yenicalisma-hayatina-hazir-misiniz.html

11. Mercer Turkey [Internet]. [cited 2022 Feb 28]: [https://www.mercer.com.tr/basin-odasihttps://www.mercer.com.tr/basin-odasi-haberler/peryon-nisan-2020-koronavirus-turkiye-arastirmasi-basin-bulteni.htmlhaberler/peryon-nisan-2020-koronavirus-turkiye-arastirmasi-basin-bulteni.html](https://www.mercer.com.tr/basin-odasi-haberler/peryon-nisan-2020-koronavirus-turkiye-arastirmasi-basin-bulteni.html)
12. Baltes BB, Briggs TE, Huff JW, Wright JA, Neuman GA. Flexible and compressed workweek schedules: A meta-analysis of their effects on work-related criteria. Journal of applied psychology. 1999; 84: 496-513. doi: 10.1037/0021-9010.84.4.496
13. Golden TD, Veiga JF, Simsek, Z. Telecommuting's differential impact on work-family conflict: Is there no place like home. Journal of applied psychology. 2005; 91: 1340-1350. doi: 10.1037/0021-9010.91.6.1340
14. Chen Y, Fulmer IS. Fine tuning what we know about employees' experience with flexible work arrangements and their job attitudes. Human Resource Management. 2018; 57: 381-395. doi: 10.1002/hrm.21849
15. Ivanauskaite A. The impact of flexible work arrangements on employee engagement and organizational commitment through the mediating role or work–family

enrichment. Unpublished Master Thesis, University of Vilnius. 2015.

16. Weideman M, Hofmeyr KB. The influence of flexible work arrangements on employee engagement: An exploratory study. SA Journal of Human Resource Management. 2020; 18: 1-18. doi: 10.4102/sajhrm.v18i0.1209
17. Richman AL, Civian JT, Shannon LL, Jeffrey Hill E, Brennan RT. The relationship of perceived flexibility, supportive work–life policies, and use of formal flexible arrangements and occasional flexibility to employee engagement and expected retention. Community, work and family. 2008; 11: 183-197. doi: 10.1080/13668800802050350
18. Zaman S, Ansari AH. Pathways to job engagement: evidences from the software industry. VINE Journal of Information and Knowledge Management Systems. 2021. doi: 10.1108/VJIKMS-06-2020-0102
19. Sonnentag S, Mojza EJ, Binnewies C, Scholl A. Being engaged at work and detached at home: A week-level study on work engagement, psychological detachment, and affect. Work

& Stress. 2008; 22: 257-276. doi: 10.1080/02678370802379440

20. Pocock B. Holding up half the sky? Women at work in the 21st century. The Economic and

Labour Relations Review. 2016; 27: 147-163. doi: 10.1177/1035304616647694

21. Almer ED, Kaplan SE. The effects of flexible work arrangements on stressors, burnout, and behavioral job outcomes in public accounting. Behavioral Research in Accounting. 2002; 14: 1–34. doi: 10.2308/bria.2002.14.1.1
22. Gajendran RS. Harrison DA. The good, the bad, and the unknown about telecommuting: Meta-analysis of psychological mediators and individual consequences. Journal of Applied Psychology. 2007; 92: 1524–1541. doi: 10.1037/0021-9010.92.6.1524
23. Glass JL, Finley A. Coverage and effectiveness of family-responsive workplace policies. Human resource management review. 2002; 12: 313-337. doi: 10.1016/S1053-

4822(02)00063-3

24. Bloom N, Liang J, Roberts J, Ying ZJ. Does working from home work? Evidence from a Chinese experiment. Quarterly Journal of Economics. 2015; 130: 165–218. doi: 10.1093/qje/qju032
25. Perry-Smith, JE, Blum TC. Work-family human resource bundles and perceived organizational performance. Academy of management Journal. 2000; 43: 1107-1117. doi: 1556339
26. Palumbo R. Let me go to the office! An investigation into the side effects of working from home on work-life balance. International Journal of Public Sector Management. 2020; 33: 771-790. doi: 10.1108/IJPSM-06-2020-0150
27. Timms C, Brough P, O'Driscoll M, Kalliath T, Siu OL, Sit C, Lo D. Flexible work arrangements, work engagement, turnover intentions and psychological health. Asia Pacific Journal of Human Resources. 2015; 53: 83-103. doi: 10.1111/1744-7941.12030
28. Bakker AB, Demerouti E. Towards a model of work engagement. Career development international. 2008; 13: 209-223. doi: 10.1108/13620430810870476
29. Smith JEP, Dumas TL. Debunking the Ideal Worker Myth: Effects of Temporal Flexibility & Family Configuration On Engagement. Academy of management proceedings Vol. 2007, No. 1: Academy of Management Annual Meeting, Briarcliff Manor; 2017. pp. 1-6. Doi:

10.5465/ambpp.2007.26508250

30. Fukuzaki T, Iwata N, Ooba S, Takeda S. Inoue, M. The Effect of Nurses’ Work–Life Balance on Work Engagement: The Adjustment Effect of Affective Commitment. Yonago Acta Medica. 2021; 64: 269-281. doi: 10.33160/yam.2021.08.005
31. Poulsen MG, Khan A, Poulsen EE, Khan SR, Poulsen AA. Work engagement in cancer care: The power of co-worker and supervisor support. European Journal of Oncology Nursing 2016; 21: 134-138. doi: 10.1016/j.ejon.2015.09.003
32. Chan CMH, Wong JE, Wee LH, Jamil NA. Yeap LLL. Swarna Nantha, Y.; Siau, C. S. Psychological and work-related factors predicting work engagement in Malaysian employees. Occupational medicine. 2020; 70: 400-406. doi: 10.1093/occmed/kqaa107
33. Aboshaiqah AE, Hamadi HY, Salem OA, Zakari NM. The work engagement of nurses in multiple hospital sectors in Saudi Arabia: a comparative study. Journal of nursing management. 2016; 24: 540-548. doi: 10.1111/jonm.12356
34. Shukla S, Adhikari B, Singh V. Employee engagement-role of demographic variables and personality factors. Amity global HRM review. 2015; 5: 65-73.
35. Abdulla, MH, Shaw JD. Personal factors and organizational commitment: Main and interactive effects in the United Arab Emirates. Journal of Managerial Issues. 1999; 77-93. doi: 40604255
36. Yildirim I. Relationships between burnout, sources of social support and Socio demographic

variables.	Social	Behavior	and	Personality.	2008;	36:	603-616.	doi:

10.2224/sbp.2008.36.5.603

37. Faskhodi AA, Siyyari M. Dimensions of work engagement and teacher burnout: A study of relations among Iranian EFL teachers. Australian Journal of Teacher Education (Online). 2018; 43: 78-93. doi: 10.14221/ajte.2018v43n1.5
38. Turunç Ö, Turgut H. Yönetim ve Strateji 101 Teori ve Yaklaşım. Ankara: Siyasal Kitabevi

Turkey; 2017.

39. Dilmaghani M. There is a time and a place for work: comparative evaluation of flexible work arrangements in Canada. International Journal of Manpower. 2020; 42: 167-192.
40. Stich JF. Flexible working and applicant attraction: a person–job fit approach. Personnel Review. 2020; 50: 358-378. doi: 10.1108/PR-12-2019-0672
41. Spreitzer GM, Cameron L, Garrett L. Alternative work arrangements: Two images of the new world of work. Annual Review of Organizational Psychology and Organizational Behavior 2017; 4: 473-499. doi: 10.1146/annurev-orgpsych-032516-113332
42. Giannikis SK, Mihail DM. Flexible work arrangements in Greece: A study of employee perceptions. The International Journal of Human Resource Management. 2011; 22: 417-432. doi: 10.1080/09585192.2011.540163
43. Abbott J, De Cieri H, Iverson RD. Costing turnover: Implications of work/family conflict at management level. Asia Pacific Journal of Human Resources. 1998; 36: 25-43. doi: 10.1177/103841119803600103
44. Timms C, Brough P, Chan XWC. Employees’ psychological health and the impact of flexible working arrangements. In Norgate SH, Cooper CL, editors. Flexible Work: Designing Our

Healthier Future Lives. Current Issues In Work And Organizational Psychology. Abingdon, UK: Routledge; 2020. pp. 35-47.

45. Kröll C, Nüesch S. The effects of flexible work practices on employee attitudes: evidence from a large-scale panel study in Germany. The International Journal of Human Resource Management. 2019; 30: 1505-1525. doi: 10.1080/09585192.2017.1289548
46. Kelliher C, Anderson D. For better or for worse? An analysis of how flexible working practices influence employees' perceptions of job quality. The International Journal of Human Resource Management. 2008; 19: 419-431. doi: 10.1080/09585190801895502
47. De Menezes LM, Kelliher C. Flexible working, individual performance, and employee attitudes: Comparing formal and informal arrangements. Human Resource Management. 2017; 56: 1051-1070. doi: 10.1002/hrm.21822
48. Eby LT, Casper WJ, Lockwood A, Bordeaux C, Brinley A. Work and family research in IO/OB: Content analysis and review of the literature (1980–2002). Journal of vocational behavior. 2005; 66: 124-197. doi: 10.1016/j.jvb.2003.11.003
49. Morelock JC, McNamara TK, James JB. Workability and requests for flexible work arrangements among older adults: The role of a time and place management intervention. Journal of Applied Gerontology. 2017; 36: 1370-1392. doi: 10.1177/0733464815624149
50. De Sivatte I, Guadamillas F. Antecedents and outcomes of implementing flexibility policies in organizations. The International Journal of Human Resource Management. 2013; 24: 1327-

1345. doi: 10.1080/09585192.2011.561225

51. Carless SA, Wintle J. Applicant attraction: The role of recruiter function, work–life balance policies and career salience. International Journal of Selection and Assessment. 2007; 15:

394-404. doi: 10.1111/j.1468-2389.2007.00398.x

52. Lee	C.	Balancing	Work	and	Family.	2022;	28:	02	Available	from:

<https://eric.ed.gov/?id=EJ430803>

53. Galinsky E, Stein PJ. The impact of human resource policies on employees: Balancing work/family life. Journal of Family Issues. 1990; 11: 368-383. doi: 10.1177/019251390011004002
54. Scandura TA, Lankau MJ. Relationships of gender, family responsibility and flexible work hours to organizational commitment and job satisfaction. Journal of Organizational Behavior:

The International Journal of Industrial, Occupational and Organizational Psychology and

Behavior. 1997; 18: 377-391. doi: 10.1002/(SICI)1099-1379(199707)18:4<377::AID-

JOB807>3.0.CO;2-1

55. Ewald A, Gilbert E, Huppatz K. Fathering and flexible working arrangements: a systematic interdisciplinary review. Journal of Family Theory & Review. 2020; 12: 27-40. doi: 10.1111/jftr.12363
56. Wickramasinghe V, Jayabandu S. Towards workplace flexibility: flexitime arrangements in Sri Lanka. Employee Relations. 2007; 29: 554-575. doi: 10.1108/01425450710826087
57. Gonsalves L. From face time to flex time: The role of physical space in worker temporal flexibility. Administrative Science Quarterly. 2020; 65: 1058-1091. doi:

10.1177/0001839220907891

58. Cech EA, Blair-Loy M. Consequences of flexibility stigma among academic scientists and engineers. Work and Occupations. 2014; 41: 86-110. doi: 10.1177/0730888413515497
59. Bakker AB, Albrecht S. Work engagement: current trends. Career Development International. 2018; 23: 4-11. doi: 10.1108/CDI-11-2017-0207
60. Kulikowski K, Sedlak P. Can you buy work engagement? The relationship between pay, fringe benefits, financial bonuses and work engagement. Current Psychology. 2020; 39: 343-

353. doi: 10.1007/s12144-017-9768-4

61. Schaufeli W, Salanova M. Work engagement: On how to better catch a slippery concept. European Journal of Work and Organizational Psychology. 2011; 20: 39-46. doi: 10.1080/1359432X.2010.515981
62. Salanova M, Schaufeli WBA cross-national study of work engagement as a mediator between job resources and proactive behaviour. The International Journal of Human Resource Management. 2008; 19: 116-131. doi: 10.1080/09585190701763982
63. Bakker AB, Schaufeli WB, Leiter MP, Taris TW. Work engagement: An emerging concept in occupational health psychology. Work & stress. 2008; 22: 187-200. doi: 10.1080/02678370802393649
64. Rayton BA, Yalabik ZY. Work engagement, psychological contract breach and job satisfaction. The International Journal of Human Resource Management. 2014; 25: 2382-

2400. doi: 10.1080/09585192.2013.876440

65. Agarwal UA. Linking justice, trust and innovative work behaviour to work engagement. Personnel Review. 2014; 43: 41-73. doi: 10.1108/PR-02-2012-0019
66. Venz L, Pundt A, Sonnentag S. What matters for work engagement? A diary study on resources and the benefits of selective optimization with compensation for state work engagement. Journal of Organizational Behavior. 2018; 39: 26-38. doi: 10.1002/job.2207
67. Kahn WA. Psychological conditions of personal engagement and disengagement at work. Academy of management journal. 1990; 33: 692-724. doi: 10.5465/256287
68. Christian MS, Garza AS, Slaughter, JE. Work engagement: a quantitative review and test of its relations with task and contextual performance. Personnel Psychology. 2011; 64: 89-136. doi: 10.1111/j.1744-6570.2010.01203.x
69. Korunka C, Kubicek B, Schaufeli WB, Hoonakker P. Work engagement and burnout: Testing the robustness of the Job Demands-Resources model. The Journal of Positive Psychology. 2009; 4: 243-255. doi: 10.1080/17439760902879976
70. Li L, Zheng X, Zhang Q. Does leaders' adoption of employee voice influence employee work engagement? Personnel Review. 2021. doi: 10.1108/PR-04-2020-0262
71. Yalabik, ZY, Popaitoon P, Chowne JA. Rayton BA. Work engagement as a mediator between employee attitudes and outcomes. The International Journal of Human Resource Management. 2013; 24: 2799-2823. doi: 10.1080/09585192.2013.763844
72. Malik P, Garg P. Learning organization and work engagement: The mediating role of employee resilience. The International Journal of Human Resource Management. 2020; 31: 1071-1094. doi: 10.1080/09585192.2017.1396549
73. Maslach C, Jackson SE, Leiter MP. Maslach burnout inventory: Third edition. In. Zalaquett,

CP, Wood RJ, editors. Evaluating stress: A book of resources. Lanham: MD: Scarecrow Press; 1997. p. 191–218.

74. Del Boca D, Oggero N, Profeta P, Rossi M. Women’s and men’s work, housework and childcare, before and during COVID-19. Review of Economics of the Household. 2020; 18:

1001-1017. doi: 10.1007/s11150-020-09502-1

75. Yaish M, Mandel H, Kristal T. Has the Economic Lockdown Following the Covid-19

Pandemic Changed the Gender Division of Labor in Israel? Gender & Society. 2021; 35: 256-

270. doi: 10.1177/08912432211001297

76. Rajesh S, Ekambaram K. The career trajectories of Indian women professionals in the IT industry: An empirical study. International Journal of Social Science and Interdisciplinary

Research. 2013; 2: 84-97.

77. Power K. The COVID-19 pandemic has increased the care burden of women and families. Sustainability: Science, Practice and Policy. 2020; 16: 67-73. doi: 10.1080/15487733.2020.1776561
78. Sonnentag S, Kruel U. Psychological detachment from work during off-job time: The role of job stressors, job involvement, and recovery-related self-efficacy. European Journal of Work and Organizational Psychology. 2006; 15: 197-217. doi: 10.1080/13594320500513939
79. Watanabe M, Yamauchi K. Subtypes of overtime work and nurses’ fatigue, mental status, and work engagement: A latent class analysis of Japanese hospital nurses. Journal of advanced nursing. 2019; 75: 2122-2132. doi: 10.1111/jan.13991
80. Walter J, Haun VC. Positive and negative work reflection, engagement and exhaustion in dual-earner couples: Exploring living with children and work-linkage as moderators. German

Journal	of	Human	Resource	Management.	2021;	35:	249-273.	doi:

10.1177/2397002220964930

81. Lundberg U, Mårdberg B, Frankenhaeuser M. The total workload of male and female white collar workers as related to age, occupational level, and number of children. Scandinavian journal of psychology. 1994; 35: 315-327. doi: 10.1111/j.1467-9450.1994.tb00956.x
82. Ten Brummelhuis, LL, Bakker AB. Staying engaged during the week: the effect of off-job activities on next day work engagement. Journal of occupational health psychology. 2012; 17: 445. doi: 10.1037/a0029213
83. Poulsen AA, Meredith P, Khan A, Henderson J, Castrisos V, Khan SR. Burnout and work engagement in occupational therapists. British Journal of Occupational Therapy. 2014; 77: 156-164. doi: 10.4276/030802214X13941036266621
84. Chung H, Van der Horst M. Women’s employment patterns after childbirth and the perceived access to and use of flexitime and teleworking. Human relations. 2018; 71: 47-72. doi: 10.1177/0018726717713828
85. Golden L. Flexible daily work schedules in US jobs: Formal introductions needed? Industrial

Relations: A Journal of Economy and Society. 2009; 48: 27–54. doi: 10.1111/j.1468-

232X.2008.00544.x

86. Sharma A, Goel A, Sengupta S. How does work engagement vary with employee demography?:—Revelations from the Indian IT industry. Procedia computer science. 2017; 122: 146-153. doi: 10.1016/j.procs.2017.11.353
87. Köse A. The Relationship between Work Engagement Behavior and Perceived

Organizational Support and Organizational Climate. Journal of Education and Practice. 2016; 7: 42-52.

88. Willmer M, Westerberg Jacobson J, Lindberg M. An exploratory analysis of work engagement among women with and without disordered eating. BMC Women's Health 2021; 21: 1-6. doi: 10.1186/s12905-021-01429-8
89. Othman N, Nasurdin AM. Job characteristics and staying engaged in work of nurses:

Empirical evidence from Malaysia. International journal of nursing sciences. 2019; 6: 432-

438. doi: 10.1016/j.ijnss.2019.09.010

90. Mayer RC, Schoorman FD. Differentiating antecedents of organizational commitment: A test of March and Simon's model. Journal of Organizational Behavior: The International Journal of Industrial, Occupational and Organizational Psychology and Behavior. 1998; 19: 15-28. doi: 10.1002/(SICI)1099-1379(199801)19:1<15::AID-JOB816>3.0.CO;2-C
91. Bamford M, Wong CA, Laschinger H. The influence of authentic leadership and areas of worklife on work engagement of registered nurses. Journal of Nursing Management. 2013;

21: 529–540. doi: 10.1111/j.1365-2834.2012.01399.x

92. Bell E, Barkhuizen N. The relationship between barriers to change and the work engagement of employees in a South African property management company. SA Journal of Industrial Psychology. 2011; 37: 01-11. doi: 10.4102/sajip.v37i1.935
93. Schaufeli WB, Salanova M, González-romá V, Bakker AB. The measurement of engagement and burnout: A two sample confirmatory factor analytic approach. Journal of Happiness Studies. 2002; 3: 71–92. doi: 10.1023/A:1015630930326
94. Dang HAH, Nguyen CV. Gender inequality during the COVID-19 pandemic: Income, expenditure, savings, and job loss. World Development. 2021; 140: 1-10. doi:

10.1016/j.worlddev.2020.105296

# Supporting Files

**S1 Fig.** **Research Model**

**S2 Table 2. T-Test Results on the Relationship between Marital Status and Work Engagement**

**S3 Table 3. ANOVA Test Results on the Relationship between Job Experience and Work**

**Engagement**

**S4 Table 4. T-Test Results on the Relationship of Having a Child and Work Engagement**

Supporting Information - Compressed/ZIP File Archive

[Click here to access/download](https://www.editorialmanager.com/pone/download.aspx?id=30838166&guid=e95712ec-3fe8-4dd9-b081-193138186540&scheme=1)

[Supporting Information - Compressed/ZIP File Archive Figures and Tables.zip](https://www.editorialmanager.com/pone/download.aspx?id=30838166&guid=e95712ec-3fe8-4dd9-b081-193138186540&scheme=1)

Data Review URL

[Click here to download Data Review UR](http://figshare.com/s/631d0c6cb9299ea95bdc)

[L](http://figshare.com/s/631d0c6cb9299ea95bdc)

[http://figshare.com/s/631d0c6cb9299ea95bd](http://figshare.com/s/631d0c6cb9299ea95bdc)

[c](http://figshare.com/s/631d0c6cb9299ea95bdc)
